# Supplementary material for: Stealthy Player in Lipid Experiments? EDTA Binding to Phosphatidylcholine Membranes Probed by Simulations and Monolayer Experiments
Source: J Phys Chem B. 2023 Jun 12;127(24):5462–9. doi: 10.1021/acs.jpcb.3c03207 (PMC10291544; doi:10.1021/acs.jpcb.3c03207)
Supplement: Supplementary file 1 — jp3c03207_si_001.pdf [file jp3c03207_si_001.pdf]

# A Stealthy Player in Lipid Experiments? EDTA Binding to Phosphatidylcholine Membranes Probed by Simulations and Monolayer Experiments

Katarina Vazdar[1,a], Carmelo Tempra[2,a], Agnieszka Olżyńska[1], Denys Biriukov[2,3], Lukasz Cwiklik[1,2,*] and Mario Vazdar[4,*]

[1]J. Heyrovský Institute of Physical Chemistry, Czech Academy of Sciences, Dolejškova 3, 18223 Prague, Czech Republic

[2]Institute of Organic Chemistry and Biochemistry of the Czech Academy of Sciences, Flemingovo náměstí 542/2, 16000 Prague, Czech Republic

[3]Central European Institute of Technology, Masaryk University, Kamenice 5, 625 00 Brno, Czech Republic

[4]Department of Mathematics, Informatics and Cybernetics, University of Chemistry and Technology, Technická 5, 16628 Prague, Czech Republic

a) K. V. and C. T. contributed equally to this work.

Corresponding authors' e-mails: lukasz.cwiklik@jh-inst.cas.cz; mario.vazdar@vscht.cz

## Supporting Information

Page 2. Composition of studied systems in MD simulations.

Page 3. Atomic types and partial charges of $EDTA^{2-}$ and $EDTA^{3-}$ anions.

Page 4. An example snapshot from EDTA-containing MD simulations.

Page 5. Simulation model of EDTA anions.

Page 6. RDFs of selected systems.

Page 7. Kinetics of POPC monolayer surface pressure changes.

| **Table S1.** Composition of all studied systems in molecular dynamics simulations. | | | | | | | |
|---|---|---|---|---|---|---|---|
| Name | POPC | $EDTA^{2-}$ | $EDTA^{3-}$ | $Na^{+}$ | $Ca^{2+}$ | $Cl^{-}$ | Water |
| 1 $EDTA^{2-}$ / 10 POPC | 512 | 51 | 0 | 102 | 0 | 0 | 41380 |
| 1 $EDTA^{3-}$ / 10 POPC | 512 | 0 | 51 | 153 | 0 | 0 | 41360 |
| 1 $Ca^{2+}$/ 0 $EDTA^{2-}$/ 10 POPC | 512 | 0 | 0 | 0 | 51 | 102 | 41380 |
| 1 $Ca^{2+}$/ 1 $EDTA^{2-}$/ 10 POPC | 512 | 51 | 0 | 0 | 51 | 0 | 41380 |
| 1 $Ca^{2+}$/ 10 $EDTA^{2-}$/ 10 POPC | 512 | 512 | 0 | 922 | 51 | 0 | 40458 |
| 1 $Ca^{2+}$/ 1 $EDTA^{3-}$/ 10 POPC | 512 | 0 | 51 | 51 | 51 | 0 | 41357 |
| 1 $Ca^{2+}$/ 10 $EDTA^{3-}$/ 10 POPC | 512 | 0 | 512 | 1434 | 51 | 0 | 40248 |

**Table S2.** Atom types and atomic partial charges for EDTA anions as assigned by CHARMM general force field, see Figure S2 for atomic name assignment. All hydrogens that are not explicitly labeled correspond to atom name H. $C_d$, $CC_d$, and $OC_d$ atoms refer only to $EDTA^{3-}$ protonation form.

| Atom name | Atom type $EDTA^{2-}$ | Charge $EDTA^{2-}$ | Atom type $EDTA^{3-}$ | Charge $EDTA^{3-}$ |
|---|---|---|---|---|
| N1 | NG3P1 | -0.134 | NG301 | -0.716 |
| N2 | NG3P1 | -0.134 | NG3P1 | -0.148 |
| C1 | CG324 | -0.050 | CG321 | 0.108 |
| C2 | CG324 | -0.050 | CG324 | -0.034 |
| H | HGA2 | 0.090 | HGA2 | 0.090 |
| $H_p$ | HGP2 | 0.270 | HGP2 | 0.270 |
| C | CG324 | -0.086 | CG324 | -0.086 |
| CC | CG2O3 | 0.603 | CG2O3 | 0.603 |
| OC | OG2D2 | -0.665 | OG2D2 | -0.665 |
| $C_d$ | - | - | CG321 | -0.011 |
| $CC_d$ | - | - | CG2O3 | 0.564 |
| $OC_d$ | - | - | OG2D2 | -0.760 |

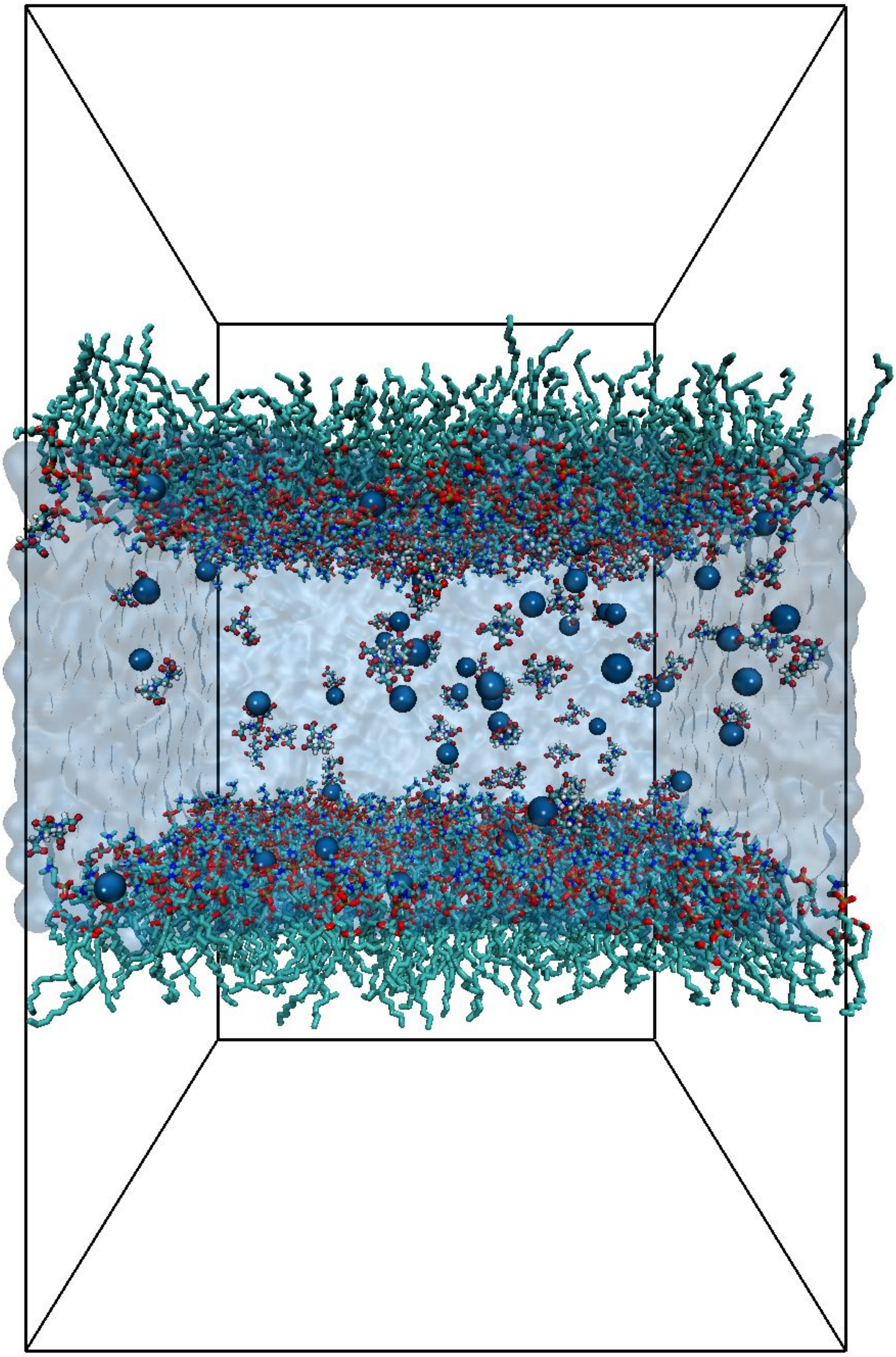

**Figure S1.** An example snapshot from MD simulations for 1 $Ca^{2+}$ / 1 $EDTA^{2-}$ / 10 POPC.

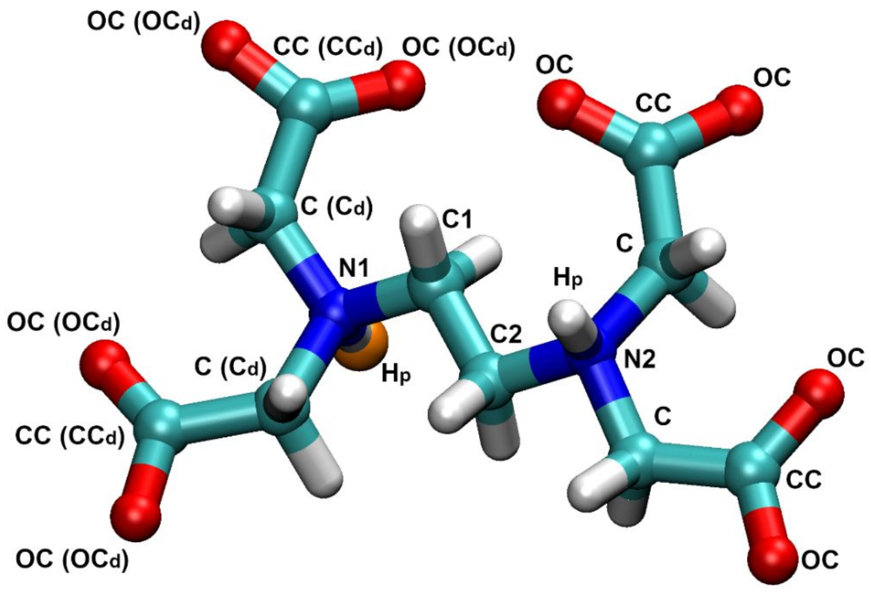


**Figure S2.** An MD model of EDTA anions. The structure of $EDTA^{2-}$ is shown, while $EDTA^{3-}$ is achieved by removing one of the protons shown as an enlarged orange sphere.

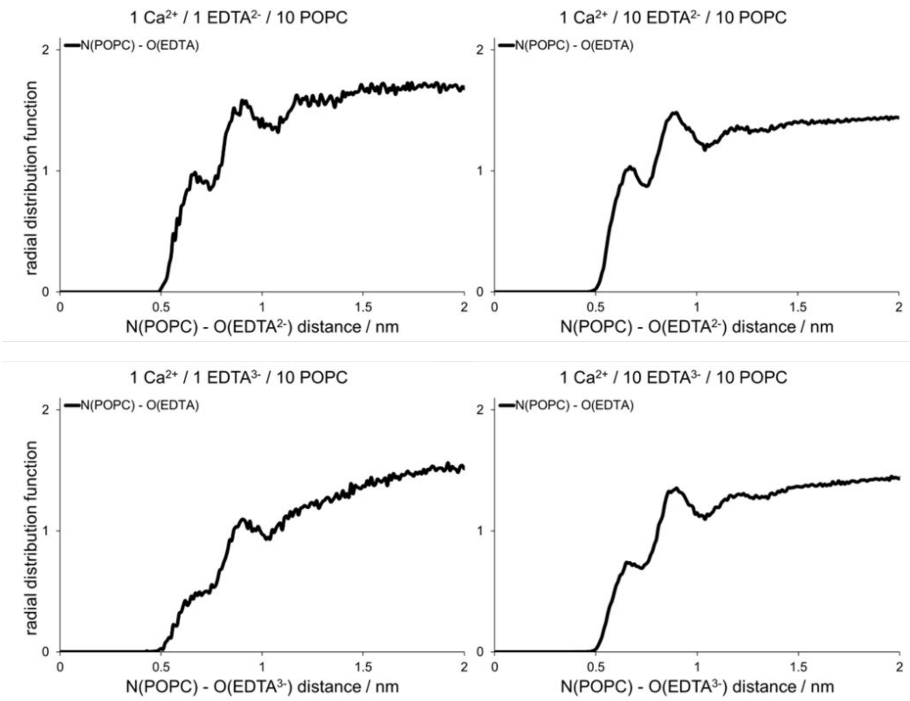


**Figure S3.** Radial distribution functions between the nitrogen atom in the POPC choline group (N(POPC)) vs. any carboxyl oxygen atom in $EDTA^{2-}$ and $EDTA^{3-}$ anion in the EDTA-containing systems.

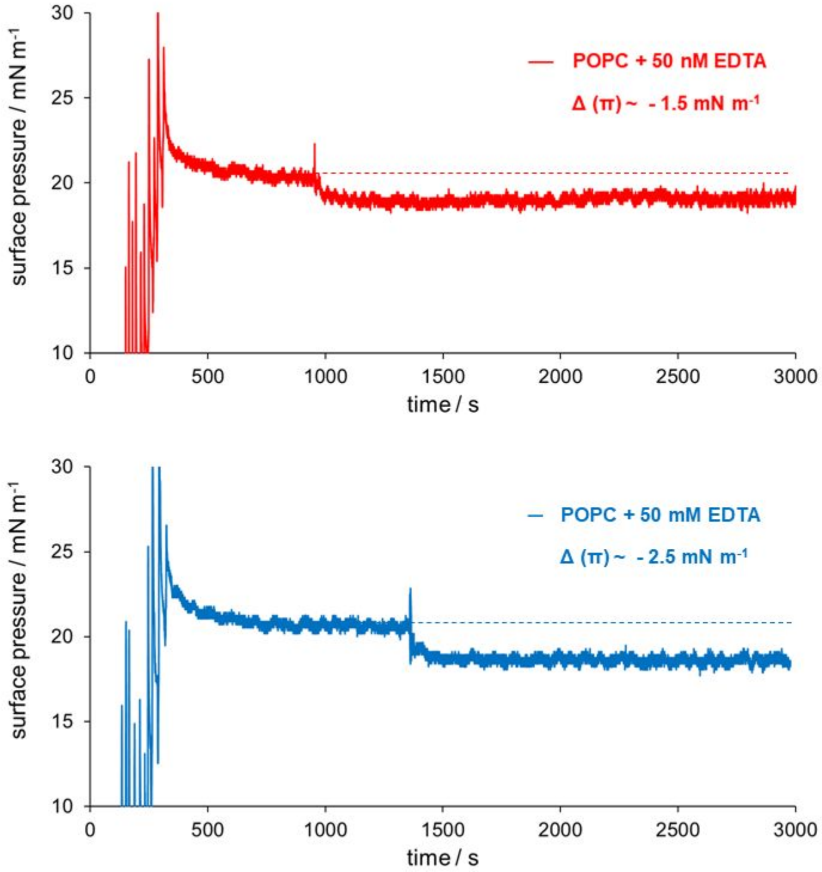


**Figure S4**. Kinetics of POPC monolayer surface pressure changes before and after the addition of EDTA using cone Langmuir trough setup with NIMA sensor. The final concentration in the solution is 50 nM at pH = 5.5 (blue, upper panel) and 50 $\mu$M at pH = 6.4 (red, bottom panel).
